# Exploring global efforts in mental health screening and early detection

**DOI:** 10.3389/fpsyt.2026.1824068

**Published:** 2026-05-20

**Authors:** Haya S. Zedan, Abeer Alshehri, Mohammed M. J. Alqahtani, Hind Alharbi, Rayan Al-Saab, Yazeed Alosimi, Abdulhameed AlHabeeb

**Affiliations:** 1 Department of Public Health, College of Health Sciences, Saudi Electronic University, Riyadh, Saudi Arabia; 2 Department of Psychology, College of Education, King Khalid University, Abha, Asir, Saudi Arabia; 3 King Khalid University, Abha, Asir, Saudi Arabia; 4 National Center for Mental Health Promotion, Riyadh, Saudi Arabia

**Keywords:** assessment, early detection, mental health, psychometry, screening

## Abstract

**Introduction:**

Screening tools are frequently used to assess symptoms of mental disorders in different settings before they worsen. They may theoretically be used in large-scale programs to support implementation of national action plans and mental health strategies in consideration of broader or more specific lines of action for support of individuals struggling with mental health issues. Although screening tools and programs are generally effective, there are inconsistencies in their design, planning, application, measurement of outcomes, mechanisms used for follow-up, and ethical considerations. These inconsistencies highlight the need for benchmarking efforts to guide the identification, assessment, and development of the most appropriate screening tools and early detection programs.

**Methods:**

A scoping review of literature related to mental health screening and early detection program frameworks, standards, protocols, practices, tools, techniques from around the world and evidence of effectiveness.

**Results:**

Of the 2213 potential studies identified, 38 were selected for inclusion. The majority were from the USA (26.3%). Most studies utilized non-randomized study designs, and there was a wide range of settings, sample sizes, life stages, and mental disorders assessed. The most commonly used tools were the Patient Health Questionnaire (PHQ), Kessler Scale, ProfScreen, and Generalized Anxiety Disorder assessment (GAD). Screening tools were found generally effective, but sensitivity and specificity varied. The Posttraumatic Symptoms Scale (PTSS-10), AC-OK, and Adolescent Psychotic-Like Symptom Screener (APSS) showed high diagnostic performance (98%, 96%, and 68%, respectively). Challenges faced included ease of use, additional resource requirements, and technological accessibility. Limited studies performed cost-effectiveness analyses.

**Conclusion:**

No single mental health screening tool is universally superior; each tool has advantages and limitations. The effectiveness of these tools depends on the population, settings, application, and integration with other care pathways.

**Recommendations:**

Future studies should include in-depth evaluations of screening tools and cost-effectiveness analyses to facilitate better comparison for diverse populations and settings, as well as the design and development of appropriate tools and programs for screening and early detection.

## Introduction

Mental disorders are among the leading causes of morbidity and disability. These include anxiety disorders, depressive disorders, bipolar disorder, autism spectrum disorders, schizophrenia, conduct disorder, idiopathic developmental intellectual disability, eating disorders, and other mental disorders (1). Mental disorders are also strongly associated with many other communicable and non-communicable diseases, intentional and unintentional injuries (2). Economic estimates and epidemiological studies have indicated that the global prevalence and incidence rates of mental disorders are considerably high and significantly impact human health and societal welfare (3). The OECD has recently issued a report to reflect on these issues on mental health system benchmarks and policy frameworks to address economic costs of mental health conditions, which includes recommendations for preventive measures (4).

Mental disorders have high prevalence among children and adolescents (aged 5–24 years) and affect 293 million or 1 in 10 individuals globally. Furthermore, around one-fifth of all disease-related disability is associated with mental disorders (5). Studies have shown strong correlations between mental health and adverse experiences in childhood and the development of mental health issues in adulthood (6–8). Mental disorders account for 31.14 million years lived with disability, which is 20.27% of all-cause disability (5). In 2021, the highest rate of age-standardized disability-adjusted life years was 8706.11, which was observed in Central Sub-Saharan Africa, while the lowest rate of 3340.99 was reported in East Asia (9). Unfortunately, a significant number of individuals remain undiagnosed due to systemic gaps in early detection.

A systematic review in 2021 assessed the evidence on economic evaluations and cost-effectiveness of mental health prevention and promotion interventions (10). Mental health screening and early detection are crucial preventive measures for identifying potential issues before they become severe. Mental health screening and early detection are essential components of public health strategies for improving mental health outcomes and ensuring timely and effective care. However, there are many challenges in the utilization of screening tools in different settings, populations, and evaluation methods.

The concept of mental health screening involves the use of brief and validated tools for the identification of individuals who may have symptoms of mental health disorders (11). Numerous screening tools are frequently used in various settings. Examples include the Daily Health Observation Scheme, ProfScreen for screening children and adolescents with mental disorders, Usage Rating Profile (URP-A), Assessment Rating Profile-Revised (ARP-A), Patient Health Questionnaire (PHQ), Reynolds Adolescent Depression Scale-2 (RADS-2), Generalized Anxiety Disorder assessment (GAD-7) in school settings (12–18), Brief Mental Health Disorder Screening Questionnaires for public safety personnel, General Health Questionnaires (GHQ-12 and GHQ-28), the Depression Anxiety Stress Scale (DASS-21), Workplace Outcome Suite (WOS), the five-item World Health Organization (WHO-5) Well-Being Index for workplaces (11, 19–22), Kessler Screening Scale for psychological distress (K6), Mental Health Inventory (MIV-5) for mood and anxiety, and the Warwick–Edinburgh Mental Well-Being Scale (WEMWBS) for communities (23–25). Certain screening tools have been effectively used In primary healthcare facilities, such as the Edinburgh Postnatal Depression Scale and the Mini-International Neuropsychiatric Interview (26, 27).

These screening tools are frequently used in different settings, and in many cases, they can detect the onset of mental disorders before symptoms worsen. Therefore, they may theoretically be used in large-scale screening and early detection programs. The development of mental health screening programs can be helpful in implementing national action plans and mental health strategies while considering broader or more specific lines of action for the early detection of individuals with mental disorders (28).

The WHO has issued a Comprehensive Mental Health Action Plan (29) and A Short Guide to Screening Programs (30), and numerous screening programs have been implemented in countries around the world. For example, Improving Access to Psychological Therapies (IAPT) is used for screening individuals with depression and anxiety in the UK (31). Similarly, in Australia, Headspace Initiatives have also been found to be effective in the early detection of mental disorders in youth (32). In schools, universal screening programs have been used to effectively identify youth with mental disorders (33). Artificial intelligence (AI) is also increasingly being integrated into screening programs and used to improve early detection of individuals with mental disorders (34).

Although these screening tools and programs are generally used effectively for screening, there are inconsistencies in their design, planning, application, measurement of outcomes, mechanisms used for follow-up, and ethical considerations. These issues highlight the need for benchmarking efforts to guide the identification, assessment, development and implementation of more appropriate program frameworks, standards, protocols, practices, and screening tools used for early detection of mental disorders (35).

## Aim and objectives

We aimed to review published research on mental health screening and early detection in primary care, schools, and workplace settings. The specific objectives of this study were to:

1. Identify program frameworks, standards, protocols, practices, tools, and techniques used for mental health screening and early detection around the world.
2. Assess evidence of their effectiveness in terms of early detection rates, access to care, cost, and overall mental health outcomes.
3. Provide recommendations for further research, development, and implementation of mental health screening for healthcare providers, administrators, educators, employers and policymakers.

## Methodology

A scoping review was performed on literature related to mental health screening and early detection program frameworks, standards, protocols, practices, tools, techniques and evidence of effectiveness from countries and regions around the world. The study was performed according to guidance issued for this type of review (36).

### Database search

Several electronic databases were selected to search for literature published in the past 30 years (1995–2025) in the English language (PubMed, Scopus, ScienceDirect, and the Cochrane Library).

### Search strategy

The selected English language databases were searched using the PRISMA guidelines for the most relevant literature using the following keywords: “mental health” OR “psychological well-being” OR “mental disorder” OR “mental illness” OR “mental stress” AND “early detection” OR “early intervention” OR “early diagnosis” OR “screening” AND “practice” OR “global standards” AND “primary school” OR “school” OR “colleges” OR “universities” OR “workplace.” Boolean operator words (AND, OR) were used to combine and refine the search terms. The search strategy and syntax were adjusted to comply with the specific requirements of each database and website (**Table 1**). The date of the last search was in June 2025. As a scoping review, PROSPERO registration was not required.

**Table 1:** Database search strategy.

Databases	Search terms
PubMed	(“mental health”[MeSH Terms] OR “psychological well-being”[MeSH Terms] OR “mental disorder”[All Fields] OR “mental illness”[All Fields] OR “mental stress”[All Fields]) AND (“early detection”[All Fields] OR “early intervention”[All Fields]) AND (“early diagnosis”[MeSH Terms] OR “early detection”[All Fields] OR “early intervention”[All Fields]) AND (“primary school”[All Fields] OR “school”[All Fields] OR “colleges”[All Fields] OR “universities”[MeSH Terms] OR “workplace”[MeSH Terms])
The Cochrane Library	((“mental health” OR “psychological well-being” OR “mental disorder” OR “mental illness” OR “mental stress”)): ti,ab,kw AND ((“early diagnosis” OR “early detection” OR “early intervention”)): ti,ab,kw AND ((“primary school” OR “school” OR “colleges” OR “universities” OR “workplace”)): ti,ab,kw
Scopus	(“mental health” OR “psychological well-being” OR “mental disorder” OR “mental illness” OR “mental stress”) AND (“early diagnosis” OR “early detection” OR “early intervention”) AND (“primary school” OR “school” OR “colleges” OR “universities” OR “workplace”)
ScienceDirect	(“mental health” OR “psychological well-being”) AND (“early diagnosis” OR “early detection” OR “early intervention”) AND (“primary school” OR “school” OR “universities” OR “workplace”)

### Screening

Studies were screened in four phases by two independent reviewers with adherence to the PRISMA guidelines (37). In the first phase, studies were identified from the electronic databases and moved to EndNote X9 referencing software for the removal of duplicates. In the second phase, studies were screened by the two reviewers based on the title and abstract, and irrelevant studies were excluded after discussion and inter-rater agreement for various reasons. In the third phase, full-text assessment was performed, and studies were selected based on the inclusion criteria. In the fourth stage, the final selected studies were included in the review for further analysis.

### Inclusion and exclusion criteria

The eligibility criteria were determined using the PCC (Population, Concept, Context) framework. Eligible studies were on mental health screening and early detection program frameworks, standards, protocols, practices, tools and techniques used for any or all age groups and mental disorders, provided evidence of effectiveness (high diagnostic accuracy, sensitivity, specificity, cost-effectiveness, impact on health outcomes), and were conducted in primary care, schools/universities, workplaces, or other relevant settings using either randomized or non-randomized study designs (cross-sectional, observational, case control, prospective, and experimental or case studies) from countries around the world.

We selected a search period of 30 years (1995–2025) to ensure retrieval of comprehensive knowledge and evidence on mental health screening practices and incorporation of technological advancements in the field. We excluded studies that were not related to mental health screening and early detection, studies that focused solely on diagnosis or early intervention without addressing screening and early detection, and studies not published in English.

### Methodological quality assessment

The included studies were assessed for quality using the Cochrane Risk of bias-2–0 tool for randomized studies, and each study was evaluated in five domains: randomization process, deviation from intended intervention, missing outcomes, outcome measurement, and selection of reported results. Non-randomized studies were evaluated using Risk of Bias in Non-Randomized Studies – Intervention (ROBINS-I) for bias due to confounding, selection of the participants, classification of interventions, deviation from intended interventions, missing data, measurement of outcomes, and selection of the reported results. Each study was rated as “low,” “some concerns,” or “high/serious risk of bias” in assessments by two independent reviewers. The outcomes of the quality assessment exercise were visualized using the ROBVIS web-based application (38).

### Data extraction, analysis and synthesis

Data were extracted from included studies using a pre-defined Microsoft Excel form while considering the following variables: study characteristics (e.g., authors, year, country, methodology, sample), participant characteristics (e.g., life stage, age, gender, comorbidities), setting (e.g., primary care, schools, workplaces, other), screening tools and techniques used, implementation process, outcomes (e.g., efficacy, sensitivity, specificity, early detection rates, access to care, mental health outcomes), challenges and barriers encountered in implementation, and data on cost-effectiveness. Quantitative findings were synthesized through statistical aggregation using contextual or categorical data, descriptive statistics and tabulation (sums, counts, minimum/maximum, percentages) where applicable; no formal quantitative synthesis or meta-analysis was performed. Qualitative findings underwent thematic analysis; grouping study findings in consideration of the characteristics of the screening tools and outcomes; reporting measures, rates of detection and diagnostic accuracy, utilization of technology or artificial intelligence, issues with access to care, challenges or barriers to implementation, and evidence on cost-effectiveness. Due to the heterogeneity of the studies included, the findings were then synthesized narratively by two reviewers to generate insight into the frameworks, tools, and techniques used. Tables were constructed to provide an overview of the characteristics of the included studies.

## Findings

**Figure 1:**
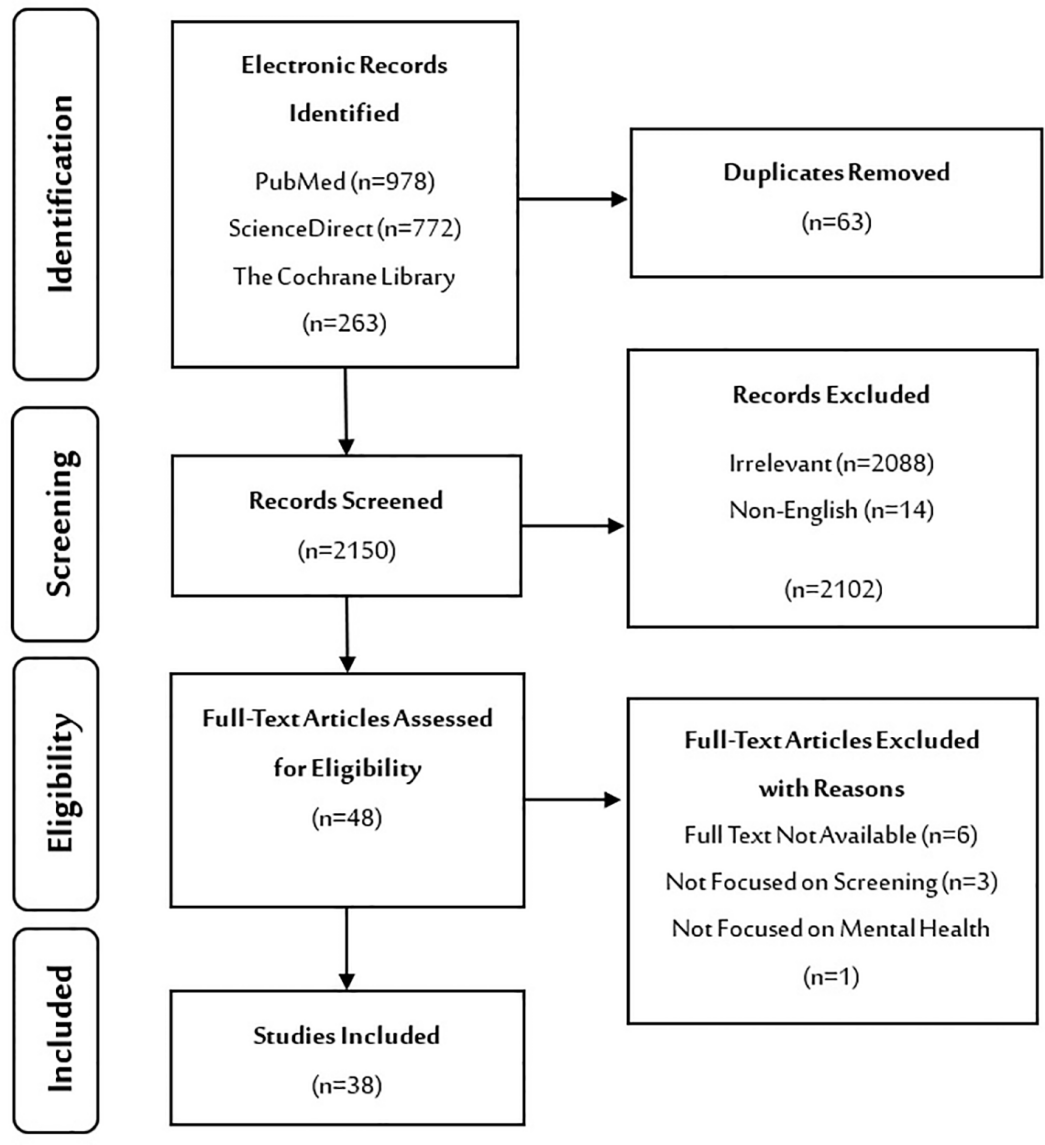
PRISMA flowchart.

In the first phase, 2213 studies were identified from the selected electronic databases and moved to the EndNote X9 referencing software, where 63 duplicate studies were removed. In the second phase, 2150 studies were screened based on the title and abstract. 2102 irrelevant studies were then excluded for various reasons. In the third phase, full-text assessment was performed on 48 studies, and another 10 studies were excluded for more specific reasons. In the last phase, the inclusion criteria were applied, and 38 studies were selected to be included in the review for analysis (**Figure 1**, PRISMA Flowchart).

## General characteristics of studies and participants

**Figure 2:**
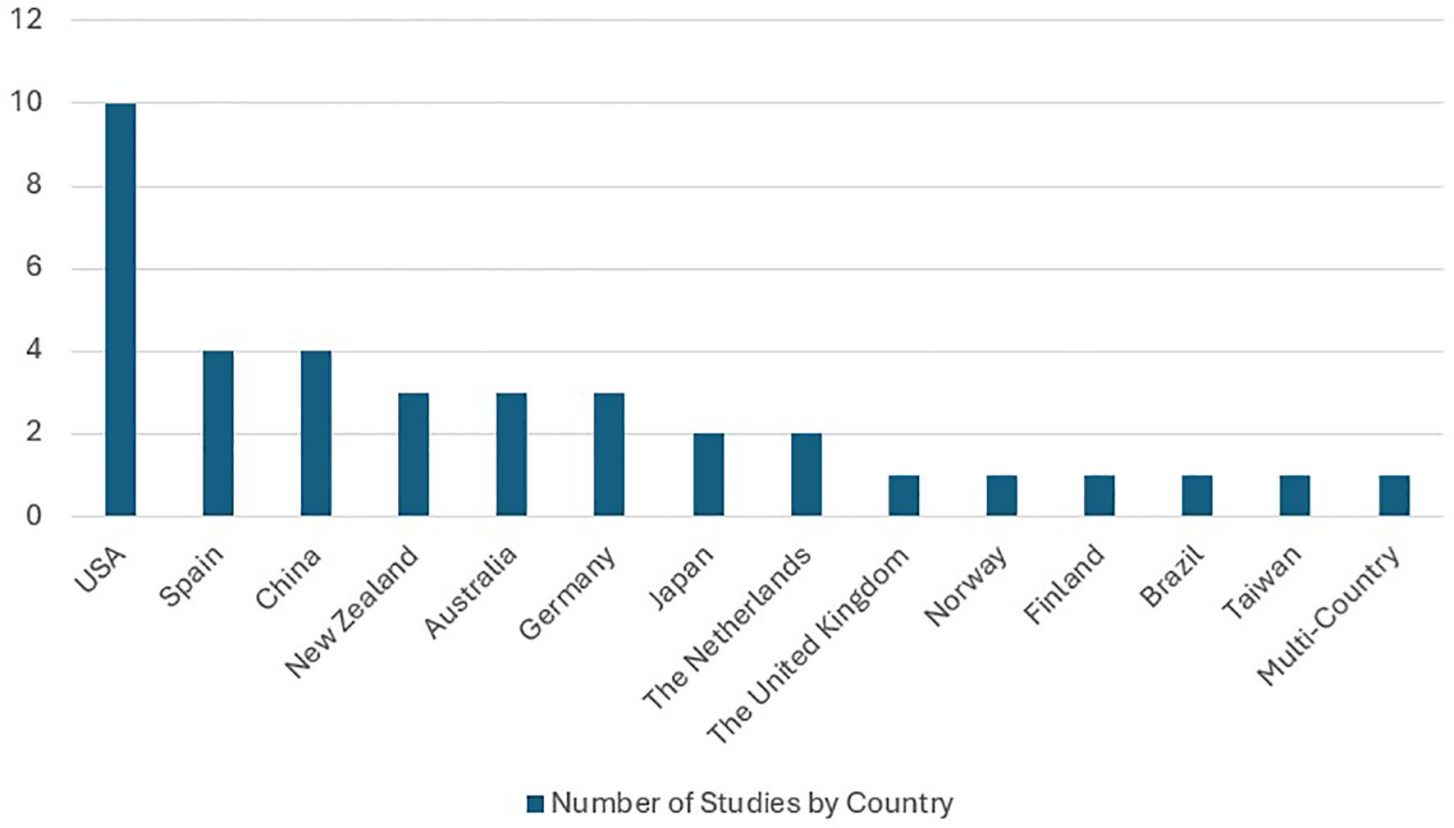
Number of studies by country.

Most of the studies (10/38, 26.31%) were reported from the USA (39–48). Four studies (10.52%) were from Spain (49–52) and China (53–56), three studies (7.89%) were from New Zealand (57–59), Australia (60–62), and Germany (13, 63, 64), two studies (5.26%) were from Japan (12, 65) and the Netherlands (66, 67), one study (2.63%) each was from the UK (68), Norway (69), Ireland (70), Finland (71), Brazil (72), and Taiwan (73), and one study (2.63%) involved multiple countries (74) (see **Figure 2**).

Most of the included studies used non-randomized study designs (cross-sectional, observational, case control, prospective, and experimental), and only four studies used randomized designs (13, 46, 50, 63) (**Table 2**). Sample sizes varied from 52 to 14,915 participants (56, 64). Most of the screening tools and programs were used in schools and university settings, followed by primary healthcare centers, community centers, and epidemiological centers (**Table 2**). The included studies spanned all life stages: adults (21/38, 55.26%), adolescents (11/38, 28.94%), children (4/38, 10.52%), and pediatrics and adolescents (1/38, 2.63%). Variation was also observed in participants’ age: the minimum age was 4.5 years (57), while the maximum age was >65 years (71). We found only limited studies reporting on comorbidities, such as acute respiratory distress syndrome (ARDS) and insomnia (64, 73(. Psychological issues that participants faced are listed in **Table 2**.

**Table 2:** Summary of general characteristics of studies and participants.

Study characteristics					Participant characteristics				
Study ID	Country	Methodology	Sample size	Study setting	Life stage	Age (years)	Gender (M:F)	Comorbidities	Psychological issue
(Murphy et al., 1996) (46)	USA	Cross-Sectional	379	Clinics	Children	10.9	203:176	NA	Psychosocial Problems
(Stoll et al., 1999) (65)	Germany	Cohort	52	Hospital	Adult	36.5	26:26	ARDS	PTSD
(MaGPIe Research Group, 2005) (59)	New Zealand	Cross-Sectional	775	Primary Care	Adult	>18	NA	NA	Mental Health
(Larsen et al., 2006) (70)	Norway	Quasi-experimental	281	Health Centers	Adult	NA	NA	NA	First-Episode Psychosis
(Priest et al., 2008) (62)	Australia	Cross-Sectional	2142	Primary Care	Adult	31.2	0:2142	NA	Psychosis
(Essex et al., 2009) (42)	USA	Cross-Sectional	Parent=879	School	Children	NA	NA	NA	Mental Health
(Zauszniewski and Suresky, 2010) (49)	USA	Cross-Sectional	60	Community	Adult	46.28	0:0.60	NA	Serious Mental Illness Relatives
(Stallman, 2010) (63)	Australia	Prospective	6479	University	Adult	18–>45	2294:4185	NA	Depression and Anxiety
(Kelleher et al., 2011) (71)	Ireland	Prospective	231	Public School	Adolescents	11–13	NA	NA	Psychotic-Like Experiences
(Kline et al., 2012) (43)	USA	Cross-Sectional	194	Community Clinics, Psychiatric Inpatient Treatment Unit	Adolescents	16.72	47:0.147	NA	Psychosis-Risk Syndromes
(Kaess et al., 2013) (73)	Austria, Estonia, France, Germany, Hungary, Ireland, Israel, Italy, Romania, Slovenia, Spain	Cross-Sectional	3070	School	Adolescents	<18	1301:1752	NA	Mental Health
(Simeonova et al., 2014) (48)	USA	Prospective	122	University	Adolescents	14.25	68:0.54	NA	Social And Behavioral Problems
(Ma et al., 2015) (54)	China	Case study	2181	Epidemiological Centers	Adult	<30–64	502:1979	NA	Depression and Other Mental Illness
(Oromendia et al., 2015) (53)	Spain	Cross-sectional	171	Community	Adult	36.33	67:104	NA	Panic Disorder
(Burakevych et al., 2016) (58)	New Zealand	Prospective	274	Pre-School	Children	4.5	138:136	NA	Emotional Health
(Eisner et al., 2017) (69)	UK	Prospective	In-Depth Interview Checklist =123, Case Note =187	Mental Health Trust	Adult	Interview Checklist =34.4 Case Note =45	Interview Checklist =11:12 Case Note =116:71	NA	Non-Affective Psychosis, Schizophrenia, Schizoaffective Disorder
(Chavez et al., 2017) (41)	USA	Cross-sectional	567	Primary Health Care	Adult	18–>50	216:351	NA	Depression, Anxiety, PTSD
(Morales-Hidalgo et al., 2017) (52)	Spain	Descriptive survey	2660	School	Children	3–12	NA	NA	Autism Spectrum Disorder and Social Pragmatic Communication Disorder
(McArdle and Lambie, 2018) (60)	New Zealand	Cross-sectional	204	Secure care facilities	Adolescents	15.3	179:0.25	NA	Mental Health
(Lopez et al., 2018) (44)	USA	Cross-sectional	319	Primary Health Care	Adult	18–>65	NA	NA	Depression
(Wu et al., 2019) (74)	Taiwan	Cross-sectional	3982	Psychiatric Outpatient, Medical Outpatients and Community	Adult	49.7	1842:2140	Insomnia	Suicide Risk Assessment, Depression, Anxiety
(Brodey et al., 2019) (40)	USA	Cross-sectional	353	Early Psychosis Sites	Adult	20.5	NA	NA	Psychosis-Risk Syndromes
(Zhang et al., 2019) (56)	China	Cross-sectional	461	University	Adult	19	209:252	NA	Chronic Stress
(Bonet et al., 2020) (50)	Spain	Cross-sectional	59	First Episode Psychosis Program	Adult	32.8	66:0.24	NA	Early Psychosis
(Brathwaite et al., 2020) (73)	Brazil	Cross-sectional	1928	School	Adolescents	14–16	976:952	NA	Depression
(Mashio and Kawaguchi, 2020) (66)	Japan	Cohort study	151	University	Adult	>18	95:0.56	NA	Anxiety, Dysphoria
(Yang et al., 2021) (55)	China	Case study	60	Hospital	Adult	18–55	26:0.34	NA	Depression
(Asare et al., 2021) (72)	Finland	Observational	629	Multi-Countries Community	Adult	18–>65	546:69	NA	Depression
(Schick et al., 2022) (64)	Germany	RCT	146	Research Lab	Adult	24.2	79:67	NA	Psychological Distress
(Fonseca-Pedrero et al., 2023) (51)	Spain	RCT	2235	Secondary Schools	Adolescents	14.49	1053:1182	NA	Anxiety Symptoms, Emotional, Behavioral
(Aalbers et al., 2023) (67)	Netherlands	Prospective	224	College Students	Adolescents	21.97	99:125	NA	Stress
(Lustig et al., 2023) (13)	Germany	RCT	4172	School	Adolescents	15	2750:1422	NA	Mental Health Problems and Risk-Behavior
(Niendam et al., 2023) (47)	USA	RCT	Intervention =2432, Control =2455	Clinics And Schools	Adolescents	17.09	NA	NA	Psychosis
(Haque et al., 2023) (61)	Australia	Experimental	476	Datasets	Pediatric Adolescent	NA	NA	NA	OCD, SAD, ADHD
(Zhang et al., 2023) (57)	China	Experimental	14915	Medical Students	Adult	32.98	5813:9102	NA	Schizophrenia, Depression, Anxiety, Other
(Rood et al., 2023) (68)	Netherlands	Cross-sectional	235	Community	Adult	31.98	52:183	NA	BDD
(Mansoor and Ansari, 2024) (45)	USA	Observational	NA	Social Media Community	NA	NA	NA	NA	Mental health
(Nishimura et al., 2024) (12)	Japan	Cross-sectional	2143	Public Schools	Adolescents	NA	1084:1059	NA	Depression and anxiety

OCD, Obsessive Compulsive Disorder; SAD, Separation Anxiety Disorder; ADHD, Attention Deficit Hyperactivity Disorder; BDD, Body Dysmorphic Disorder; NA, Not Available; USA, United States of America; UK, United Kingdom; M; Male; F; Female; ARDS, Acute Respiratory Distress Syndrome; PTSD, Post-Traumatic Stress Disorder.

## Characteristics of screening tools and outcomes

### Commonly used screening tools and reporting methods

There was significant variation in the usage of screening tools for mental disorders among the included studies. The most commonly used tools were the PHQ (12, 43, 50, 71), Kessler Scale (62, 63), ProfScreen (13, 74), and GAD (12). The responses to most of these tools were self-reported except for a few, which were reported by parents and teachers (12, 41, 45–47, 51, 57, 70).

### Effectiveness, rates of detection and diagnostic performance

Overall, screening tools were found to be effective, but two studies reported screening tools as ineffective due to low sensitivity (30%) and accuracy (62%): the General Health Questionnaire (GHQ-12) and the Mini-International Neuropsychiatric Interview for Children and Adolescents (MINI-KID) (58, 72). Sensitivity and specificity varied, and tools like the Posttraumatic Symptoms Scale (PTSS-10) and Adolescent Psychotic-Like Symptom Screener (APSS) showed high diagnostic performance (**Table 3**). Screening tools such as the PTSS, AC-OK, and the questionnaires based on the Diagnostic and Statistical Manual of Mental Disorders (DSM) demonstrated high early detection rates of 98%, 96%, and 68%, respectively (40, 41, 64).

**Table 3:** Summary of characteristics of mental health screening tools and outcomes.

Study ID	Screening tool characteristics		Outcomes								
	Screening tool used	Implementation process	Efficacy	Sensitivity	Specificity	Early detection rate	Access to care	Mental health outcome	Challenges	Cost-effectiveness	Conclusions
(Murphy et al., 1996) (46)	PSC	Parent Reported	Effective	NA	NA	0.50–2.9%	Children Need More Care	NA	NA	NA	PSC Well-Accepted Screening Tool
(Stoll et al., 1999) (65)	PTSS-10	Self-Reported	Effective	77%	97.50%	98%	NA	Significant # Required PTSD Treatment	NA	NA	PTSS-10 Responsive, Valid, Reliable Screening Tool
(MaGPIe Research Group, 2005) (59)	GHQ-12	Self-Reported	Not Effective	30%	94.50%	5/100 Cases	NA	NA	NA	NA	Sensitivity Suggests Not Appropriate Tool
(Larsen et al., 2006) (70)	Early Detection Program	Self-Reported	Effective	NA	NA	64%	NA	NA	NA	NA	Early Detection Program Possible and Important Despite No Obvious Effect on Positive Symptoms During First Year of Treatment
(Priest et al., 2008) (62)	PRAM	Self-Reported	Effective	NA	NA	5.30%	Easily	NA	NA	Minimum Number of Medical Staff Required	Offers Conceptual Framework for Brief Psychosocial Assessment
(Essex et al., 2009) (42)	DSM Ontario Child Health Study Adult-Report MacArthur Health Behavior	Parent and Teacher Reported	Effectively Identified	81%	95%	Kindergarten=19% Grade 1 = 21% Grade 3 =27% Grade 5 = 16%	NA	NA	NA	NA	School Entry Screening Can Effectively Identify Children
(Zauszniewski and Suresky, 2010) (49)	DCS	Self-Reported	Effective	NA	NA	68%	NA	NA	NA	NA	DCS Found Useful for Early Detection of Depression in Women Relatives of Adults with Serious Mental Illness
(Stallman, 2010) (63)	Kessler 10	Self-Reported	Effective	NA	NA	University Students had Higher Stress Levels	Need More Care	NA	Only Focused on Anxiety and Depression	NA	Effectively Screened University Students for Anxiety and Depression
(Kelleher et al., 2011) (71)	APSS	In-Depth Clinical Interview	Effective	70%	82.60%	High Detection Rate	Need More Care	NA	NA	NA	Short Questionnaires Can Be Used for Early Detection of Mental Disorders
(Kline et al., 2012) (43)	Prime Screen Prodromal Questionnaire Brief, Youth Psychosis At-Risk Questionnaire Brief	Self-Reported	All Three Screening Tools Effective	Prime Screen =80% Prodromal =95% Youth Psychosis At-Risk =65%	Prime Screen =48% Prodromal =28% Youth Psychosis At-Risk =76%	Accuracy: Prime Screen =31% Prodromal =55% Youth Psychosis At-Risk =71%	NA	NA	No Single Tool Emerged Clear Frontrunner	NA	All Three Screeners Appear to Useful and Valid Assessment Tools for Attenuated Symptoms
(Kaess et al., 2013) (73)	ProfScreen	Self-Reported	Effective	NA	NA	61%	NA	12.5%	NA	NA	Reliable Screening Tool
(Simeonova et al., 2014) 48]	CBCL	Parent Reported	Effective	NA	NA	Increased	NA	NA	NA	Cost-effective	Reliable Screening Tool
(Ma et al., 2015) (54)	Epidemiologic Studies Depression Scale	Self-Reported	Effective	NA	NA	20.60%	NA	NA	NA	Required Extra Cost and Resources	Effectively Used to Identify People with Mental Illness
(Oromendia et al., 2015) (53)	WSQ	Web-Based	Accuracy =82%	83%	74%	NA	NA	NA	Only Internet Users	NA	The WSQ-Panic Accuracy Acceptable Internet Screening Tool
(Burakevych et al., 2016) (58)	B4SC	Teacher-Reported	Effective	NA	NA	19%	NA	NA	NA	NA	B4SC Pre-School Screening Assessment Identified Children with Developmental and Emotional Health Issues
(Eisner et al., 2017) (69)	In-Depth Interviews, Verbal Checklists Basic Symptoms Case Note Extracts	Self-Reported	Effective	NA	NA	In-Depth Interview Verbal Checklist =100% Case Note =72.2%	NA	NA	NA	NA	Majority Interviewees Self-Reported Pre-Relapse Basic Symptoms
(Chavez et al., 2017) (41)	AC-OK	Interview and Self-Reported	Effective	USA=73% Spain=80%	USA=83% Spain=77%	96%	NA	NA	NA	NA	Reliable Screening Tool
(Morales-Hidalgo et al., 2017) (52)	EduTEA	Teacher-Reported	Effective 89% Accuracy	87%	91%	10%	NA	NA	NA	NA	EduTEA Useful ASD Screening Protocol in Schools
(McArdle and Lambie, 2018) (60)	MAYSI-2	Self-Reported	Effective	NA	NA	80%	NA	NA	No	Low Cost	Young People in Secure Facilities Have Substantial Service Needs
(Lopez et al., 2018) (44)	PHQ-9	Self-Reported	Effective	NA	NA	33%	NA	NA	NA	NA	Reliable Screening Tool
(Wu et al., 2019) (74)	CMHC-9	Self-Reported	Effective	92%	82%	High Predict for Recent Suicidal Ideation	NA	NA	NA	NA	CMHC-9 Brief and Effective Tool for Suicide Risk Detection
(Brodey et al., 2019) (40)	SIPS, EPSI-SR	Self-Reported	PPV: EPSI = 76.5% SIPS=68.5%	EPSI =47.1% SIPS=100%	EPSI =77.4% SIPS=0%	EPSI Misses 49% of Current or Future Psychotic Cases	NA	NA	Low Sensitivity	NA	EPSI First Validated Assessment to Predict 12-Month Psychotic Conversion
(Zhang et al., 2019) (56)	COPE Scales	Self-Reported	Effective	NA	NA	25%	NA	NA	NA	NA	COPE Identify Students with Highest Correlation Between Coping Behavior and Mental Health
(Bonet et al., 2020) (50)	ReMindCare App	Mobile App	Effective	NA	NA	NA	NA	20%	NA	NA	App Proved Benefits in Real-World Treatment of Early Psychosis
(Brathwaite et al., 2020) (73)	MINI-KID	Self-Reported	Not Effective 62% Accuracy	NA	NA	NA	NA	NA	NA	NA	Poorer Overall Performance Compared to Original Brazilian Cohort
(Mashio and Kawaguchi, 2020) (66)	Anxiety and Dysphoria Scale	Self-Reported	Effective	NA	NA	Significant Detection of High-Risk Group	NA	NA	NA	NA	Effectively Used to Identify Students with Mental Illness
(Yang et al., 2021) (55)	Facial Expression	Technology Based	Accuracy: TOFS = 78% LBP=55% HOG=61% MDMO=65%CNNs=60%	NA	NA	Successful Detection of Early Cases	NA	NA	NA	NA	Model Effective Reflect Feasibility and Validity of Prototype
(Asare et al., 2021) (72)	PHQ-8	Self-Reported	Effective	NA	NA	16.81%	Easily	NA	NA	NA	Traditional Assessment of Depression Augmented with Behavioral Markers from Smartphones for Depression Diagnosis and Monitoring
(Schick et al., 2022) (64)	Kessler-18	Paper-Based Chatbot Web-Based	Chatbot Comparable Outcomes	NA	NA	Chatbot Significant in Detecting Cases	NA	Validation Study	Chatbots Difficult to Use	NA	Chatbot Valid Outcomes Can Be Used As E-Health
(Fonseca-Pedrero et al., 2023) (51)	PHQ-9	Self-Reported	Adequate Goodness of Fit Indices	NA	NA	Effective Early Detection Depression Symptoms	NA	NA	NA	NA	Brief Easy Reliable Tool
(Aalbers et al., 2023) (67)	Smartphone-Tracked Digital Stress Markers	Self-Reported	Effective	NA	NA	55.3%	NA	NA	NA	NA	Smartphone Log Data Can Be Used for Identifying Digital Markers of Stress
(Lustig et al., 2023) (13)	ProfScreen	Self-Reported	Effective	NA	NA	40.7%	10.1%	NA	NA	NA	Effectively Used to Identify People with Mental Illness
(Niendam et al., 2023) (47)	Technology-Enhanced Screening	Telephone Interview	Effective	NA	NA	5.60%	13	NA	NA	NA	Effectively to Identify People with Mental Illness
(Haque et al., 2023) (61)	Tree-Based Pipeline Optimization Tool	Machine Learning Algorithm	Effective Accuracy: OCD = 91% SAD=79% ADHD= 91%	NA	OCD=96%SAD=91% ADHD =99%	NA	NA	Validation Study	NA	NA	App Found Effective in Detecting Mental Illnesses Early
(Zhang et al., 2023) (57)	MRI	MIL	Effective 76% Accuracy	77%	74%	MIL=84% Self Report =22%	NA	NA	NA	NA	Model Identified Individual Serious Mental Illness Patients with Good Accuracy and High Sensitivity
(Rood et al., 2023) (68)	BDDS-5	Self-reported	Effective	NA	NA	55 Participants Found with BDD	NA	NA	NA	NA	BDDS-5 Valid Widely Applicable Screener for BDD May Help in Early Detection
(Mansoor and Ansari, 2024) (45)	AI-Powered Social Media Analysis	Social Media Post	Accuracy =89.3% Precision =86.7%	NA	NA	Suicidal Ideation =93.5% Depressive Episodes =91.2% Manic Episodes =88.7% Anxiety Crises =87.3%	NA	NA	Limited to Only Publicly Available Posts	NA	AI-Powered Social Media Potential for Early Detection of Mental Health Crises
(Nishimura et al., 2024) (12)	PHQ-4 Based on PHQ-9 GAD-7 Based on Item from PHQ-2	Teacher-Reported	Effective	NA	NA	NA	NA	NA	NA	NA	School-Based Mental Health Screening Is Feasible When Data from Daily Health Observations Can Be Used Through Digitization

PSC, Pediatric Symptom Checklist; GAD-7, Generalized Anxiety Disorder-7; PHQ, Patient Health Questionnaire-4; AI, Artificial Intelligence, BDDS-5, Body Dysmorphic Disorder Screener for DSM-5; MRI, Magnetic Resonance Imaging; CNNs, convolutional neural networks; TOFS, Transitional Optical Flow under Stimulus; LBP, Local Binary Pattern; HOG, Histograms of Oriented Gradients, MINI-KID, Mini International Neuropsychiatric Interview for Children and Adolescents; SIPS, Structured Interview for Psychosis-risk Syndromes, EPSI-SR, The Early Psychosis Screener for Internet-SR; PPV, Positive Predictive Value, CMHC ,  9-item Concise Mental Health Checklist, MAYSI-2, Massachusetts youth screening instrument – second version; B4SC, Before School Check; CBCL, Child Behavior Checklist; DCS, The Depressive Cognition Scale, PTSS-10, The modified German version of the Post-Traumatic Stress Syndrome 10-Questions Inventory; PTSD, Post-Traumatic Stress Disorder; PRAM, Psychosocial Risk Assessment Model; APSS, Adolescent Psychotic-Like Symptom Screener, GHQ-12, General Health Questionnaire; WSQ, Web Screening Questionnaire; MIL, Multiple Instance Learning; NA, Not Available.

### The use of technology and artificial intelligence

Emerging technologies such as smartphone-tracked stress markers, AI-powered social media analysis, and machine learning algorithms have also demonstrated potential for accurate screening of mental disorders (44, 49, 54, 60).

### Access to care and cost-effectiveness

Only a few studies emphasized the need for enhanced care following positive results for mental disorders. Challenges faced by these screening tools included ease of use, additional resource requirements, and technological accessibility (**Table 3**). Notably, limited studies performed cost-effectiveness analyses, but screening tools and programs such as the Psychosocial Risk Assessment Model (PRAM), the Child Behavior Checklist (CBCL), and Massachusetts Youth Screening Instrument (MAYSI-2) were found to be low-cost options (47, 53, 59, 61). Overall, our findings show that no single screening tool can be considered universally superior, and each tool has advantages and limitations; the effectiveness of these tools depends on the population, settings, application, and integration with other care pathways (**Table 3**).

## Methodological quality assessment

**Figure 3:**
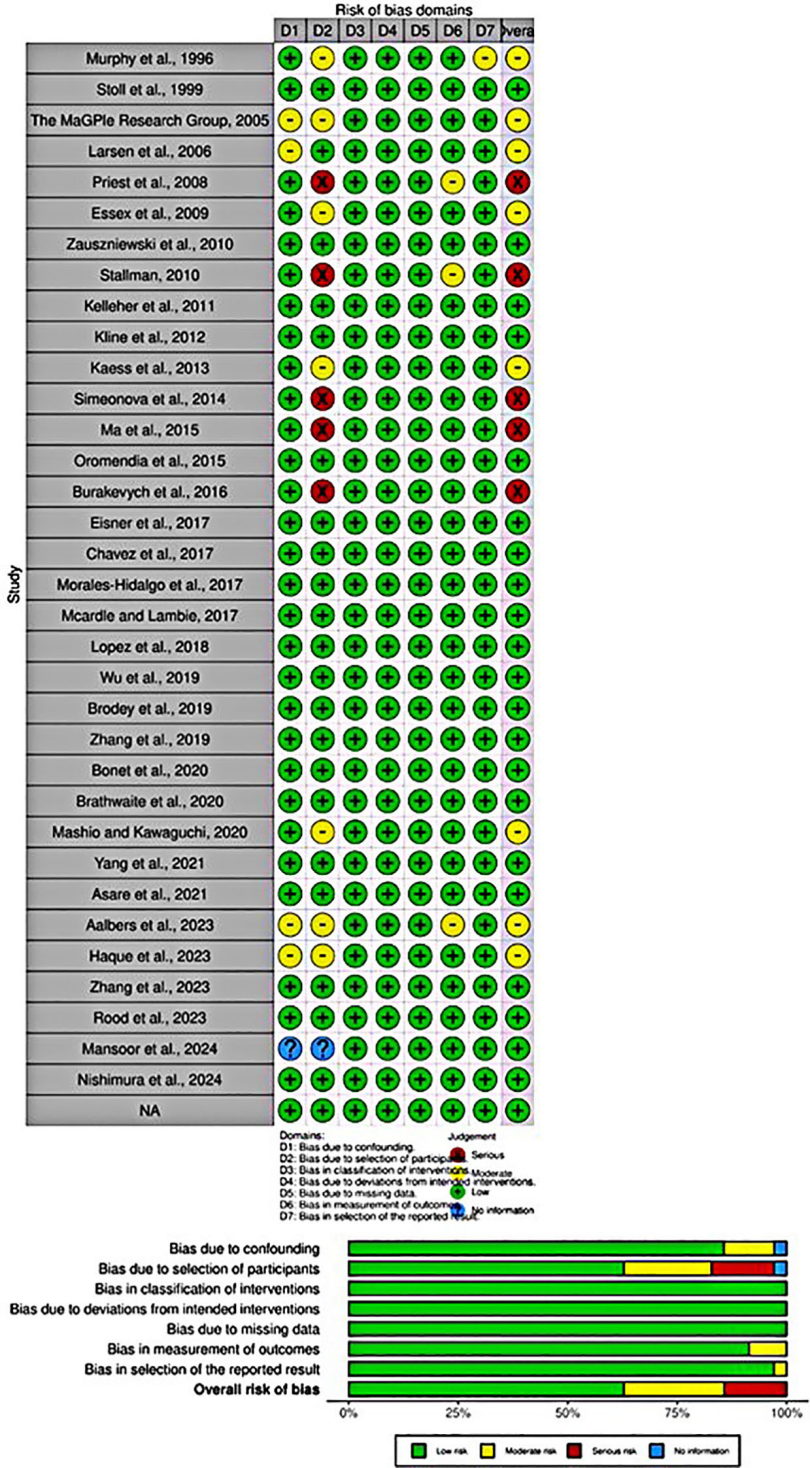
Methodological quality assessment of non-RCTs.

**Figure 4:**
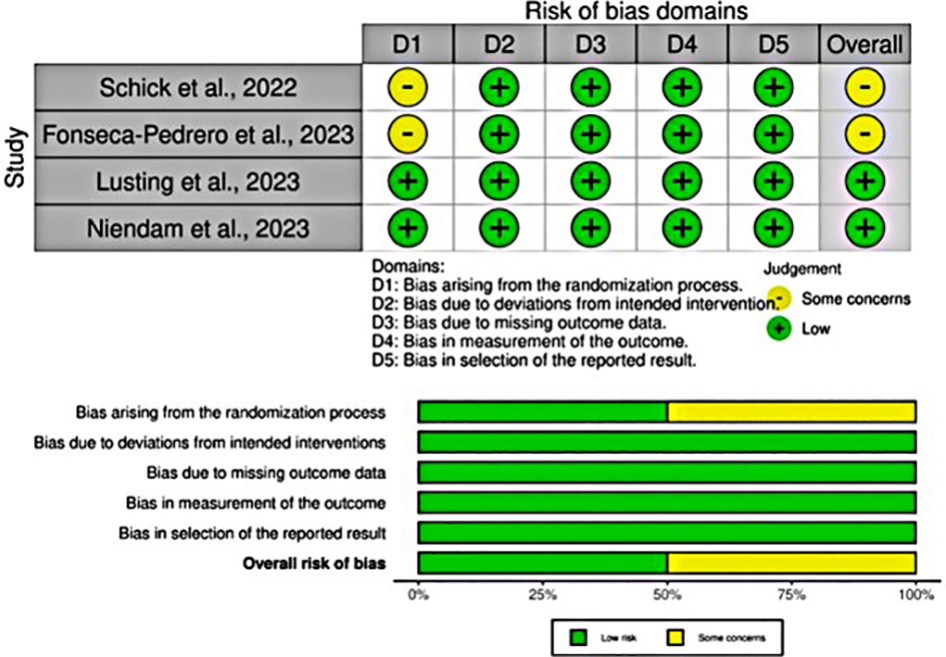
Methodological quality assessment of RCTs.

Among non-RCTs, most of the studies had a low risk of bias and no concerns in any domain. Eight studies had some issues in the domain of bias due to confounding, participant selection, outcome measurement, and outcome reporting (41, 45, 58, 60, 65, 66, 69, 74). Five studies had a high risk of bias in the domain of participant selection (47, 53, 57, 61, 62) (**Figure 3**). Among RCTs, two studies demonstrated a low risk of bias in all domains (13, 46), while two studies raised concerns about bias arising from the randomization process (50, 63) (**Figure 4**).

## Discussion

Significant variation was observed in the usage of screening tools in populations and settings, however we were able to identify the most commonly used screening tools, such as the PHQ, K-Scales, and GAD. Sensitivity and specificity also varied, with tools like PTSS-10 and APSS showing high diagnostic performance. Screening tools such as the PTSS, AC-OK, and DSM-based questionnaires demonstrated high early detection rates. Overall, all screening tools were found to be generally effective, although the effectiveness of these tools depends on the populations, settings, and integration with other care pathways. Similar findings were observed in another review on the screening of common mental disorders in different settings, including general communities, universities, and elderly populations (75).

The PHQ-9 was among the most commonly used screening tools, but due to its limitations and advantages, a broader recommendation was not made; tools can be used in specific contexts (75). The Malay version of the PHQ-9 was evaluated for screening depression and showed a sensitivity of 69% and specificity of 60.5%. It was concluded that PHQ-9 is a valid and reliable screening tool that can be used effectively in primary care centers (76). Another study demonstrated that the PHQ-9 had 86% sensitivity and 67% specificity for the diagnosis of depression symptoms in Ethiopian adults in outpatient departments (77).

In another comparative study, different screening tools like the PHQ-9, K Scale, GAD-2, and EPDS were compared for screening mental disorders in pregnant women. The K-10 and K-6 were the best performing for anxiety disorders, while the EPDS was best fit for depression (78). The GHQ and BSI screening tools have also been found to have high sensitivity (85.9% and 81.2%) and specificity (87.8% and 90.8%, respectively) (79). The selection of screening tools should be based on the setting, cultural context, and individual conditions such as anxiety, depression, stress, distress, or any other behavioral or mental disorders of interest, and attention should be given to the evidence on its efficacy in detection.

In the present review, most of the screening tools and programs for mental health disorders were used for screening children and adolescents in schools and adults in university settings. These settings provide more accessible structured and organized platforms for the early detection and screening of individuals with mental disorders. The US Preventive Services Task Force (USPSTF) recommends screening children and adolescents for anxiety symptoms (80). Students at school and university levels are in various critical developmental stages where developmental or psychological issues often emerge or intensify (81).

The screening and early detection of mental disorders in children can help to decrease the chances of childhood mental disorders developing into adult disorders (6–8). Several studies included in this review have confirmed this potential (12, 13, 50, 60, 72, 74), along with a recently conducted cross-sectional study from India, which included adolescent schoolgirls and reported a high rate (48.78%) of common mental disorders, including depression, anxiety, and distress (82). Screening may potentially facilitate the provision of timely support and may also be helpful in reducing stigma around mental health challenges by integrating mental health awareness into educational systems, which can help to improve student wellbeing and educational performance (83, 84).

Certain limitations of this review should be mentioned. The focus on English language studies should be noted. A limited number of randomized controlled trials were identified and included, which could be due to challenges inherent in designing and implementing such trials in the field, as well as the practicality of conducting brief cross-sectional studies. Notable issues were flagged during the methodological assessment for the randomization methodology in two of the four trials included, and there were issues related to confounding, participant selection, and measurement and reporting of outcomes. Thus, the quality of the evidence should be considered accordingly. Due to the heterogeneity of the included studies, direct comparisons and meta-analyses of the findings were not performed.

The scope of this review did not include the influence of sociocultural variables on the selection, application, and efficacy of screening tools in various populations and contexts, which may in turn influence the detection of mental disorders. The paucity of findings on cost-effectiveness of screening and early detection is also worthy of note. Future studies should consider more in-depth analysis of the effectiveness of these screening tools for the detection of symptoms or mental disorders and consider elements of sensitivity, specificity, cost, and socio-cultural context and relevance. Studies should also compare these tools with the potential uses of artificial intelligence and advanced machine learning techniques.

## Conclusions

The present review provides a comprehensive overview of programs and tools used to screen for mental disorders around the world. With appreciation of the variations in populations, settings, and mental disorders assessed, significant variation in the tools used for the screening of mental disorders was found. The PHQ, K-Scales, and GAD were found to be the most commonly utilized screening tools. The PTSS-10 and APSS showed good diagnostic performance, and the PTSS, AC-OK, and DSM-based questionnaires demonstrated high early detection rates. However, none of the screening tools was considered superior; each tool was found to have advantages and limitations, as such the findings should not be interpreted as definitive evidence of optimal screening practices. Future studies should include more in-depth and comparative evaluations of screening tools and cost-effectiveness analyses to facilitate better comparison across diverse population settings.

## Data Availability

The original contributions presented in the study are included in the article/supplementary material. Further inquiries can be directed to the corresponding author.
